# TFEB-mTORC1 feedback loop in metabolism and cancer

**DOI:** 10.15698/cst2017.10.103

**Published:** 2017-10-01

**Authors:** Chiara Di Malta, Andrea Ballabio

**Affiliations:** 1 Telethon Institute of Genetics and Medicine (TIGEM), via Campi Flegrei, 34, 80078 Pozzuoli (Naples), Italy.; 2 Jan and Dan Duncan Neurological Research Institute, Houston, TX 77030, USA.; 3 Department of Molecular and Human Genetics, Baylor College of Medicine, Houston, Texas 77030, USA.; 4 Medical Genetics Unit, Department of Medical and Translational Science, Federico II University, Via Pansini 5, 80131 Naples, Italy.

**Keywords:** mTORC1, TFEB, RAGD, transcription, metabolism, cancer

Everyday most organisms face the requirement to adapt their metabolic cues to the fluctuation of nutrient resources into the environment. When nutrients are abundant cells activate anabolic pathways, such as protein and lipid synthesis, and promote cell proliferation and growth. On the contrary, when food availability is scarce or even absent, cells shut off anabolism and activate a catabolic program that guarantees their own survival through the generation of energy from the degradation of intracellular substrates 1.

A key regulator of metabolic adaptation to nutrient availability is the mTOR Complex 1 (mTORC1) kinase, which promotes anabolic and limits catabolic pathways through the phosphorylation of target substrates 2. Multiple inputs modulate mTORC1 activity, in particular amino acids are essential for its full activation. Amino acids mediate mTORC1 activation through the highly-conserved family of RagGTPases. Mammals have four types of Rag GTPases, Rag A, B, C and D, which form RagA or RagB/RagC or RagD obligate heterodimers 3. In presence of amino acids Rag proteins are activated through a GDP/GTP molecular switch that enables them to interact with the raptor subunit of mTORC1 to mediate its re-localization from the cytoplasm to the lysosome 4,5,6. mTORC1 lysosomal localization is a "sine qua non" condition for its activation, since its activator Rheb also localizes to the lysosome 7.

MiT/TFE transcription factors (TFs) are master regulators of lysosomal and melanosomal biogenesis and autophagy and their activity is inhibited by mTORC1 8,9,10,11,12,13. In presence of nutrients mTORC1 phosphorylates MiT/TFE TFs at critical serine residues, leading to their cytoplasmic retention. RagGTPases are also directly implicated in this event, since active Rags interact with MiT/TFE TFs and contribute to their lysosomal recruitment 14.

During starvation, inhibition of mTORC1 and activation of the phosphatase calcineurin lead to MiT/TFE de-phosphorylation and nuclear translocation 15. Once in the nucleus, they induce expression of a large group of genes that support cellular catabolism, such as lysosomal and autophagy genes. In this way, the cell regulates the expression of catabolic genes according to nutrient availability.

In our recent work we postulated that MiT/TFE TFs, which are regulated by mTORC1, could in turn influence mTORC1 activity. To test this hypothesis we analyzed mTORC1 signaling in cells and in tissues of mice in which the activity of MiT/TFE TFs was artificially manipulated.

We observed that overexpression of TFEB or TFE3 increased mTORC1 activation whereas their depletion significantly impaired mTORC1 signaling upon nutrient stimulation both in vitro and in vivo. Thus, the activation of mTORC1 in response to nutrients is regulated by MiT/TFE TFs.

mTORC1 promotes protein synthesis in muscles upon physical exercise to support muscle growth 16. This effect is well appreciated by bodybuilders, who know that eating a protein-rich meal after exercise is a good strategy to increase muscle mass. However the mechanisms underlying this process are still poorly understood. Interestingly, TFEB nuclear translocation is induced upon physical exercise as consequence of calcineurin phosphatase activation 15. Therefore, we hypothesized that TFEB could boost mTORC1 signaling in response to a meal after exercise. Consistently, exercised muscle-specific *TFEB* KO mice showed a reduced induction of mTORC1 activity and protein synthesis in response to leucine after exercise, indicating that mTORC1 activation upon physical exercise requires MiT/TFE TFs. Our findings suggested the existence of a new, physiologically relevant, molecular pathway, that regulates mTORC1 activity through MiT/TFE TFs.

Next, we investigated the mechanism underlying this regulation. We tested the hypothesis that MiT/TFE genes regulate the expression of genes that are important for mTORC1 activity. We searched for the presence of multiple MiT/TFE DNA binding sites, defined as "CLEAR elements", in the proximal promoters of 50 human genes encoding mTORC1-related genes.

Among the putative candidates, the RagD GTPase mRNA levels were the most significantly increased as a consequence of TFEB overexpression.* RagC* transcript levels, as well as those of *Folliculin* (*FLCN*), a GTPase activating protein (GAP) for RagC/D 17, were also increased, albeit at lower levels, whereas *RagB* and *RagA* were unchanged. Opposite effects were observed by *TFEB *or* TFE3* siRNA-mediated silencing. Chromatin immuno-Precipitation (ChiP) and luciferase assay demonstrated that *RagD* is a direct target of TFEB. Importantly, CRISPR-mediated deletion of the MiT/TFE response element in the RagD promoter suppressed RagD expression and mTORC1 activation upon amino acid stimulation. In line with the notion that Rag GTPases mediates mTORC1 lysosomal localization, we found that mTOR localization on the lysosome correlated with TFEB levels, being significantly decreased in cells lacking the *RagD* promoter region bound by MiT/TFE factors.

How modulation of RagD levels alone can be sufficient to significantly impair mTORC1 signaling remains an open question. One would expect that RagC could compensate for RagD but our study suggest that these two Rags are less interchangeable than what was previously thought. Another critical point is that to be functionally active RagD needs to form heterodimers with either RagA or B. We noticed that RagD levels are lower compared to the other Rag GTPases (data not shown), therefore, one possibility is that RagD represents a limiting factor for RagD/RagA and RagD/RagB heterodimer formation. In line with this hypothesis is the observation that perturbing MiT/TFE mediated transcriptional regulation of RagD only is sufficient to severely hampers mTORC1 lysosomal recruitment and hence its activation.

**Figure 1:**
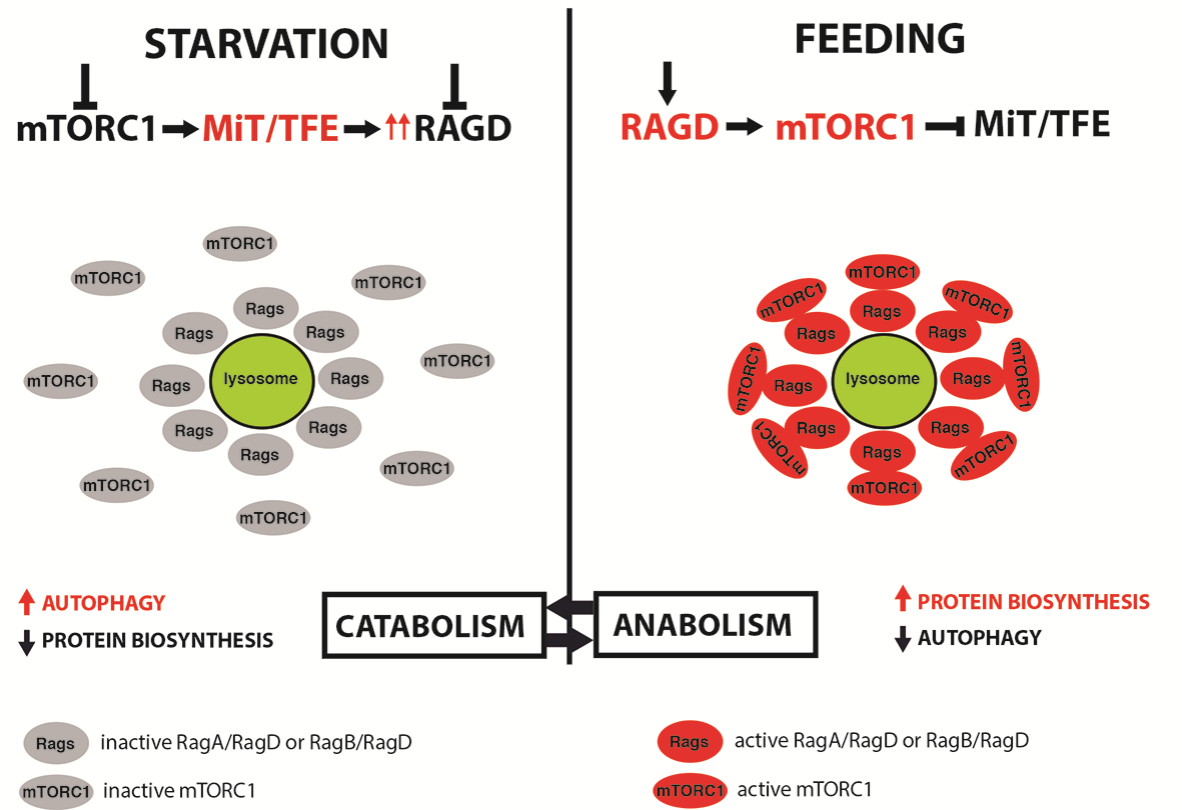
FIGURE 1: Proposed model of MiT/TFE-mediated trans¬criptional regulation of mTORC1 signaling according to nutritional status. Starvation switches off mTORC1 and MiT/TFE TFs translocate to the nucleus where they promote expression of genes involved in catabolic pathways as well as RagD GTPase, thus promoting the assembly of inactive Rags heterodimers on the lysosome. Feeding turns on RagGTPases, which can now efficiently recruit mTORC1 to the lysosome and promote its activation. This mechanism enables the cell to efficiently switch between catabolism and anabolism according nutrient availability.

In summary, we defined a new regulation of mTORC1 activity in response to nutrients. Starvation inhibits mTORC1 thus allowing MiT/TFE nuclear translocation and hence activation of catabolism via the expression of autophagic and lysosomal genes. At the same time, MiT/TFE TFs induce the expression of RagD and this results in the assembly of inactive Rags heterodimers on the lysosomal surface. Feeding turns on Rags which can now recruit mTORC1 to the lysosome and promote its activation. In this way, the cell gets ready to efficiently switch on anabolism and turn off catabolism when nutrients become available (**Fig. 1**).

Constitutive activation of MiT/TFE TFs drives tumorigenesis in several cancer types, such as Renal Cell Carcinoma (RCC), melanoma and pancreatic ductal adenocarcinoma (PDA) 18,19,20. However, the role of MiT/TFE factors in oncogenesis is still unclear. We demonstrated that constitutive induction of RagD GTPase and mTORC1 hyper-activation are hallmarks of MiT/TFE-dependent RCC, PDA and melanoma. Importantly, we found that silencing of RagD strongly reduced xenograft tumor growth of melanoma cells presenting elevated RagD expression and increased mTORC1 activation, indicating that RagD contributes to tumor cell growth in these malignancies. These findings define a new oncogenic pathway in MiT/TFE dependent tumors with potential implications for therapy.

Interestingly, clinical data indicated that patients affected by RCC caused by TFE3 chromosomal translocation frequently presented activation of mTOR signaling with variable suppression of cancer growth by treatment with mTOR inhibitors 18. Our study provides the molecular mechanism for these clinical observations and encourages further research on the potentiality of this therapeutic approach for MiT/TFE dependent malignancies.
